# Nutritional competition within tumor microenvironment dictates anti-tumor immunity

**DOI:** 10.1093/nsr/nwad277

**Published:** 2023-11-01

**Authors:** Yankang Fei, Xuetao Cao, Juan Liu

**Affiliations:** National Key Laboratory of Immunity and Inflammation, Institute of Immunology, Naval Medical University, China; National Key Laboratory of Immunity and Inflammation, Institute of Immunology, Naval Medical University, China; Department of Immunology, Chinese Academy of Medical Sciences, China; Institute of Immunology, College of Life Sciences, Nankai University, China; National Key Laboratory of Immunity and Inflammation, Institute of Immunology, Naval Medical University, China

The crosstalk between metabolism and immunity has emerged as an essential regulator of tumor immune escape, and targeting immunometabolic pathways holds great potential for cancer immunotherapy [1]. Abnormal metabolic changes in the nutrient-deficient, hypoxic, and acidic tumor microenvironment (TME) challenges the viability and function of multiple tumor-infiltrating immune cell types such as dendritic cells (DCs), macrophages, and T cells [2–4]. Therefore, it is essential to investigate how the metabolic changes in the TME impact anti-tumor immune responses, and in turn how the intracellular metabolic reprogramming in various immune cells modulate tumor progression and immunotherapy responses.

**Figure 1.**
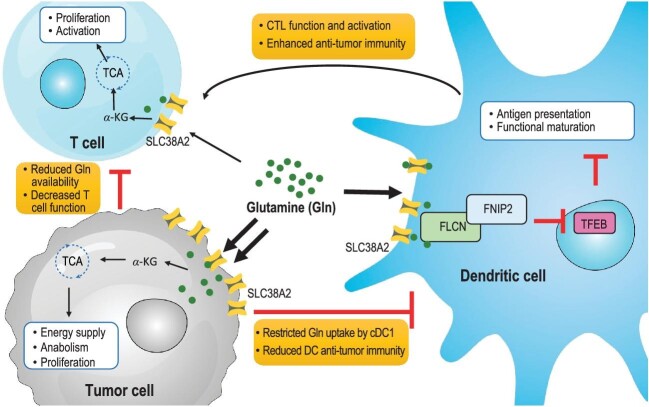
Glutamine crosstalk in the tumor microenvironment dictates tumor growth and immune escape. Glutamine is transported into tumor cells, tumor-infiltrating T cells and dendritic cells via carrier protein SLC38A2. Glutamine is metabolized by proliferating tumor cells as the main energy source to meet the TCA cycle and promote malignant growth. Dr. Hongbo Chi's group has recently demonstrated that glutamine supplementation could enhance cDC1-mediated priming of CD8^+^ T cells via regulating the FLCN/FNIP2/TFEB axis, and thereby contribute to enhanced anti-tumor immunity and improved responsiveness to immune checkpoint blockade therapy [6]. Thus, glutamine uptake and signaling serve as key checkpoint events in dictating cDC1-tumor crosstalk and putative targets for cancer immunotherapy.

Glutamine actively provides carbon and nitrogen sources for cells and plays pleiotropic roles in cancer cells via regulating cell proliferation, signaling, activation, and metastasis [5]. However, it still remains unresolved how glutamine uptake and metabolism influence DC-dependent anti-tumor immunity. It was recently reported that glutamine uptake and signaling are critical regulators of cDC1-mediated anti-tumor immunity and potential targets for cancer immunotherapy [6]. In this study, the authors demonstrated that intratumor glutamine supplementation could promote cDC1-mediated anti-tumor immunity and enhance responsiveness to immunotherapy. The glutamine/SLC38A2/FLCN axis potently orchestrates cDC1-mediated coordination of CD8^+^ T cell accumulation and function in promoting anti-tumor immunity. Moreover, high SLC38A2 expression in tumor cells restricts glutamine uptake by cDC1s, highlighting glutamine uptake via SLC38A2 as a checkpoint in dictating cDC1-tumor crosstalk. Therefore, selective promotion of glutamine uptake in DCs could serve as a combinational strategy to overcome resistance to cancer immunotherapy (Fig. 1).

Abnormal glucose metabolism in the TME leads to accumulation of various

oncometabolites, such as lactate and succinate with pro-tumor activities. Immune cells rely on glucose for functional adaptation to TME, and macrophages have recently been emphasized as the major consumer of glucose in TME [7]. Increased glucose metabolism in tumor-associated macrophages (TAMs) could fuel O-GlcNAcylation of lysosomal Cathepsin B, resulting in cancer metastasis and chemoresistance. Therefore, targeting glucose metabolism represents a promising strategy to skew the pro-tumor function of TAMs toward an anti-tumor function [8]. Metabolic partitioning in the TME also potently impacts the accumulation, activity and function of tumor-infiltrating lymphocytes [9]. One recent study showed that the SREBP2-dependent mevalonate-cholesterol pathway is the most prominent metabolic feature of tissue resident memory CD8^+^ T (TRM) cells, and that enforcing synthesis of the non-sterol products coenzyme Q can enhance memory T cell formation following viral infection and amplify anti-tumor immunity [10].

In sum, these findings uncovered the metabolic crosstalk between tumor cells and immune cells that dictates the immunosuppressive properties of TME, and revealed nutritional partitioning and competition as putative targets for cancer treatment. Further exploration of how immunometabolism is spatiotemporally remodeled in distinct TME components and how metabolism shapes complex interactions between immune and non-immune cells within the TME will lead to a more comprehensive understanding of tumor immune escape and outline new clues for development of novel cancer immunotherapies.
